# The First Norovirus Longitudinal Seroepidemiological Study From Sub-Saharan Africa Reveals High Seroprevalence of Diverse Genotypes Associated With Host Susceptibility Factors

**DOI:** 10.1093/infdis/jiy219

**Published:** 2018-04-18

**Authors:** Lucy Thorne, Angela Nalwoga, Alexander J Mentzer, Alexis de Rougemont, Myra Hosmillo, Emily Webb, Margaret Nampiija, Allan Muhwezi, Tommy Carstensen, Deepti Gurdasani, Adrian V Hill, Manj S Sandhu, Alison Elliott, Ian Goodfellow

**Affiliations:** 1 Division of Virology, Department of Pathology, University of Cambridge, UK; 2 Division of Infection and Immunity, University College London, Medical Research Council Centre for Medical Molecular Virology, UK; 3 Medical Research Council/Uganda Virus Research Institute, Uganda Research Unit, Entebbe, Uganda; 4 Wellcome Trust Centre for Human Genetics, University of Oxford, UK; 5 Centre National de Référence des Virus Entériques, Laboratoire de Virologie-Sérologie, Centre Hospitalier Universitaire de Dijon, France; 6 L'Unité Mixte de Recherche Procédés Alimentaires et Microbiologiques, Université de Bourgogne Franche-Comté/AgroSup Dijon, France; 7 Department of Infectious Disease Epidemiology, London School of Hygiene and Tropical Medicine, UK; 8 Wellcome Trust Sanger Institute, Hinxton, Cambridge, UK; 9 The Jenner Institute, University of Oxford, UK; 10 Department of Clinical Research, London School of Hygiene and Tropical Medicine, UK; 11 School of Public Health, University of Makeni, Sierra Leone

**Keywords:** norovirus, seroepidemiology, Uganda, histo-blood group antigens

## Abstract

**Background:**

Human noroviruses (HuNoVs) are a prominent cause of gastroenteritis, yet fundamental questions remain regarding epidemiology, diversity, and immunity in sub-Saharan African children. We investigated HuNoV seroprevalence and genetic and sociodemographic risk factors in Ugandan children.

**Methods:**

We randomly screened 797 participants of a longitudinal birth cohort (Entebbe, EMaBS) and 378 from a cross-sectional survey (rural Lake Victoria, LaVIISWA), for antibodies against HuNoV genotypes by ELISA. We used linear regression modeling to test for associations between HuNoV antibody levels and sociodemographic factors, and with the human susceptibility rs601338 *FUT2* secretor SNP and histo-blood group antigens (A/B/O).

**Results:**

Of EMaBS participants, 76.6% were seropositive by age 1, rising to 94.5% by age 2 years. Seroprevalence in 1 year olds of the rural LaVIISWA survey was even higher (95%). In the birth cohort, 99% of seropositive 2 year olds had responses to multiple HuNoV genotypes. We identified associations between secretor status and genogroup GII antibody levels (GII.4 *P* = 3.1 × 10^−52^), as well as ABO and GI (GI.2 *P* = 2.1 × 10^−12^).

**Conclusions:**

HuNoVs are highly prevalent in Ugandan children, indicating a substantial burden of diarrhea-associated morbidity with recurrent infections. Public health interventions, including vaccination, and increased surveillance are urgently needed.

## Teaser

This dataset provides essential insight into the high community seroprevalence of norovirus in 2 cohorts of Ugandan children, including a longitudinal birth cohort. We identify repeat infections with diverse genotypes, and social and genetic risk factors associated with HuNoV infection.

**(See the Editorial Commentary by Mans, on pages 676–8.)**

Gastroenteritis remains the second leading cause of infection-related deaths in children under 5 years old globally; sub-Saharan Africa bears the greatest burden [1]. Human noroviruses (HuNoVs) are a leading cause of viral gastroenteritis across all age groups; however, information regarding their impact and epidemiology in African children is extremely limited [2, 3].

The *Norovirus* genus of positive-strand RNA viruses is divided into 7 proposed genogroups (GI–GVII), each subdivided into genotypes, on the basis of the major capsid protein and polymerase sequences. Strains within genotype GII.4 have been responsible for the majority of outbreaks since 1996, with new pandemic variants emerging every few years, although greater diversity has been reported in some low-income settings [4].

In low- and middle-income countries, HuNoVs are responsible for >200000 deaths/year in children <5 years old [5], although this is likely to be an underestimate given the lack of surveillance in most low-resource countries. Recent studies, including 2 pioneering multicenter surveys of childhood diarrheal diseases, have begun to address the gaps in knowledge of pediatric HuNoV infections in low- and middle-income countries [6–9]. The geographical distribution of data from these and other studies highlights the paucity of information on HuNoV prevalence in sub-Saharan Africa [8–10]. The majority of studies of HuNoV in Africa have focused on incidence reporting in diarrhea cases for small cohorts using reverse transcription polymerase chain reaction (RT-PCR) based methods. Serological data can complement incidence reporting to provide an overall picture of infections in a particular population. The last study of HuNoV seroprevalence from an African country was published in 1999 [11]; since then the global prevalence of HuNoV is thought to have increased due to the emergence of pandemic strains belonging to the GII.4 genotype.

Susceptibility to HuNoV-induced diarrhea is at least partially governed by expression of histo-blood group antigens (HBGAs) in the gastrointestinal tract, which serve as viral attachment factors [12]. Intestinal HBGA expression is controlled by the α(1,2) fucosyltransferase 2 (FUT2) enzyme. Individuals homozygous for a *FUT2* nonsense mutation (G428A) are termed “nonsecretors” (Se−) and have been shown to be less susceptible to infection by certain genotypes of HuNoV, although the relationship is complex [13]. There has not yet been a large-scale extensive investigation of the association between HBGA status and HuNoV infections in an African population.

Recent breakthroughs in the development of long-sought systems for HuNoV in vitro cultivation [14, 15], mean that targeted antivirals and a multivalent vaccine for HuNoVs are within reach, with a vaccine currently advancing in phase 2b clinical trials [16]. Understanding HuNoV prevalence and natural immunity in children in sub-Saharan Africa, where any future vaccine could have the greatest impact, is therefore essential. To address this, we investigated the seroprevalence, age of seroconversion, genotype diversity, and genetic and social risk factors for HuNoVs among 2 cohorts of Ugandan children.

## METHODS

### Ethical Approval

This study was approved by the Research and Ethics Committee Uganda Virus Research Institute, Uganda National Council for Science and Technology, and London School of Hygiene and Tropical Medicine Research and Ethics Committee.

### Study Populations

The Entebbe Mother and Baby Study (EMaBS) (ISRCTN32849447, http://emabs.lshtm.ac.uk/) is a birth cohort that originated as a trial of anthelminthics [17]. In total, 2507 pregnant women in their third trimester were enrolled between 2003 and 2005. Blood samples collected from the children every year after birth (between 2004 and 2011) with informed parental consent. The study catchment area was comprised periurban, fishing, and rural communities around Entebbe and Lake Victoria. From this cohort, 797 children were randomly selected for this study. The Lake Victoria Island Intervention Study on Worms and Allergy-related diseases (LaVIISWA), included a baseline survey performed in 2012–2013 among 26 island fishing communities in Koome subcounty, Mukono District, Uganda [18]. All available samples from 1–5 year olds in the LaVIISWA baseline survey were studied.

### Virus-Like Particle Preparation

Virus-like particles (VLPs), which are structurally and antigenically indistinguishable from native virions, were generated for each strain as previously published [19]. Strains from different genotypes, some associated with pediatric infections [20, 21], were selected to represent a diverse range that would detect responses to recent and older strains. As the genotype GII.4 displays the greatest antigenic variability and rates of evolution [22, 23], we selected a GII.4 strain that was identified prior to the study sampling dates (Hu/GII.4/FRA/Dijon/1996/Dijon171, AF472623). Non-GII.4 genotypes are more antigenically static [21, 23] and the following strains were selected: GI.1 (Hu/NV/I/Norwalk/68/US, GenBank accession no. AAA59229), GI.2 (Hu/GI.2/FRA/Dijon/2008/E2818, GenBank accession no. KP064095), GII.3 (Hu/GII.3/ FRA/Dijon/2008/E2419, GenBank accession no. KP064097), GII.6 (Hu/GII.6/FRA/Dijon/2011/E5915, GenBank accession no. KP064098), and GII.12 (Hu/GII.12/FRA/Dijon/2010/E5152, GenBank accession no. KP064099).

### ELISA for HuNoV IgG Levels

To determine IgG responses to a pool of HuNoV VLPs, enzyme-linked immunosorbent assays (ELISAs) were performed as previously reported [19] (Supplementary Methods). For the EMaBS cohort, we began by screening plasma samples from children aged 1 year; any that tested negative were then followed up at each subsequent age group until a positive response was detected. We estimated the cumulative seroprevalence for each year group, making the assumption that the missing samples seroconverted at the same rate as the rest of the age group (Supplementary Methods). To screen for individual genotype responses, we randomly selected another 800 seropositive samples from 2-year-old children.

### Statistical Analysis

To estimate the cumulative seroprevalence in the EMaBS cohort (Supplementary Table 2) for ages 2 years and onwards; we made the assumption that children whose samples were not available at follow-up ages would have seroconverted at the same rate as the ones for whom samples were available in that age group. Therefore we calculated the rate of seroconversion for each age group (as number seropositive/number available to screen) and multiplied it by the total number that should have been screened for that age group (the number that had tested seronegative at the previous age) to give the estimated positive number. This value was added to the estimated positive values of each previous year and expressed as a percentage out of the total number of children included in the study (n = 797). HuNoV seroprevalence and 95% confidence intervals for the LaVIISWA survey were obtained using “svy” commands in Stata.

 Linear regression modeling of risk factors was performed using Stata-13 software (Stata 13.0, Statacorp, College Station). For both EMaBS and LaVIISWA, IgG levels (OD450 values) to HuNoV were log10 transformed to become approximately normally distributed. For EMaBS, linear regression with bootstrapped confidence intervals was used to determine the association between HuNoV antibodies and various risk factors: sex, geographical location, mother’s occupation, household socioeconomic status (hhSES), human immunodeficiency virus (HIV) exposure status, and diarrhea events. Mother’s occupation was categorized into 3 groups based on relatedness of occupation. Group 1 included farming/fishing, unskilled manual labor, and bar/hotel attendants; group 2 included housewives; while group 3 included business, students, and professionals. A composite value was obtained for hhSES derived from the home building materials, number of rooms, and items collectively owned at the mother’s home, using principal component analysis. Geographical location was categorized into 3 groups: urban (Entebbe), periurban (Kingungu and Manyago), and rural (Katabi). The mother’s HIV status was used to group children as either exposed or unexposed, as the number of children that tested positive for HIV at 1 year was very low (7/773). For mother’s occupation and geographical location, the groups were analyzed as nominal variables after testing for departure from linear trend. In the crude analysis we reported the *P* value from the Wald test and in the adjusted analysis we reported the *P* value from the likelihood ratio test.

For LaVIISWA, linear regression analyses employed the “svy” survey commands in Stata to allow for clustering of respondents within villages using linearized standard errors and for variable village sizes using weights. Exposures considered were sex, age, and child’s school status. Because the outcome variable (IgG antibody levels to HuNoV) was log transformed prior to linear regression modeling, geometric mean ratios were derived by back-transforming regression coefficients and their corresponding 95% confidence intervals (CI). During univariate analysis, each risk factor was analyzed separately with the outcome variable for each study. A linear trend assumption was made and tested using the likelihood ratio test when analyzing age and hhSES, mother’s occupation, and geographical location. During multivariate analysis all risk factors were analyzed with the outcome variable using 1 model for each study.

Associations between secretor status and ABO phenotypic blood group were assessed using the Student *t* test implemented in R. Differences in log-transformed distributions of genotype-specific antibody levels were tested between 2 groups and the final *P* value reported for each comparison. To test for evidence of interaction between ABO and *FUT2* loci, the likelihood of an interaction model was compared to the likelihood of a multivariable model (with no interaction) including secretor status (Se+ or Se−) and blood group (O or B), using the likelihood ratio test. Sensitivity analyses were performed to ensure that the statistics generated from the genetic association analyses were not false positives originating from population stratification (Supplementary Methods). Associations between genetic variants and diarrhea incidence were assessed using Cox regression with robust standard errors to allow for the fact that some children experienced multiple diarrhea episodes.

### Genotype Data for Determining FUT2 Secretor Status and ABO Blood Type

Whole-genome genotype data were available for 651/797 selected children from EMaBS generated on the Illumina Omni2.5M array and quality controlled using standard pipelines, including removing individuals and variants with high levels of missingness or deviations from expected levels of heterozygosity or Hardy-Weinberg equilibrium. Untyped genetic variants, including the *FUT2* secretor status SNP (rs601338 at position 49206674 on chromosome 19 in genome build 37) and *ABO* SNPs (rs8176747 position 136131315 and rs8176719 position 136132908 on chromosome 9) were imputed using a merged 1000 Genomes and African-specific reference panel [24] using SHAPEIT2 [25] and IMPUTE2 [26] with default settings applicable for African populations, and all imputed variants were filtered using an info score threshold of 0.3 and a minor allele frequency of 0.01 or greater. Imputed dosages of secretor A alleles were used to determine secretor status. Se status was confirmed by PCR amplification and Sanger sequencing of the rs601338 SNP at position 49206674 on chromosome 19 for 80 randomly selected EMaBS participants. For ABO, we used phased genotypes to determine genetic blood groups using Supplementary Table 1 (adapted from [27]). The corresponding phenotypic blood groups used for downstream analyses were determined using these genetic blood groups.

## RESULTS

**Figure 1.**
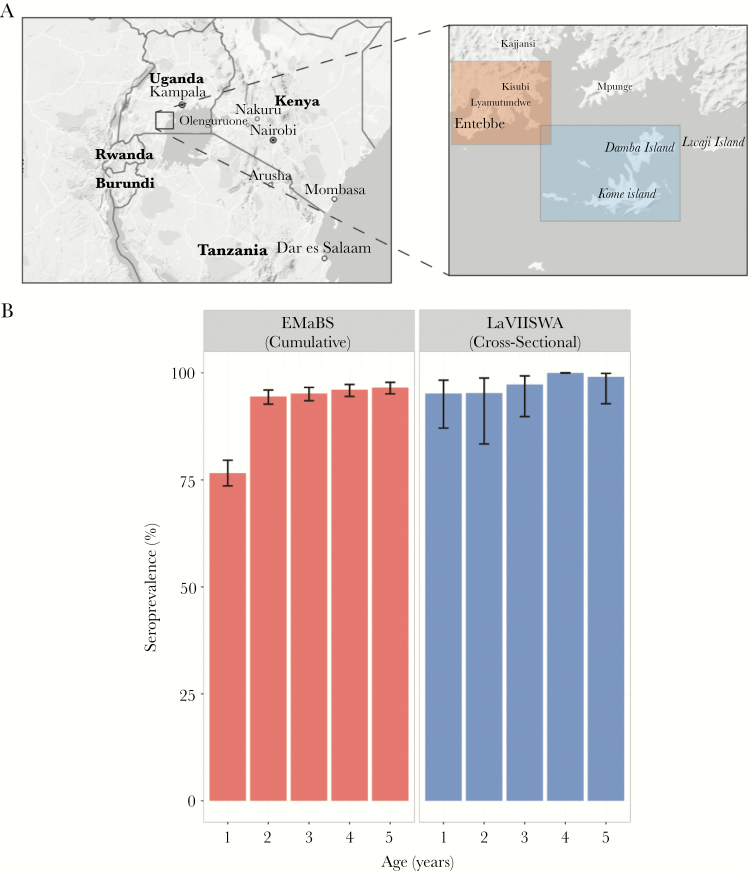
Human norovirus (HuNoV) seroprevalence in Ugandan children between 1 and 5 years old. *A*, Study catchment areas for the longitudinal birth cohort around Entebbe (Entebbe Mother and Baby Study [EMaBS], shown in red) and a cross-sectional study in the islands of Lake Victoria (Lake Victoria Island Intervention Study on Worms and Allergy-related diseases [LaVIISWA] , shown in blue). *B*, Cumulative (EMaBS) and cross-sectional (LaVIISWA) seroprevalence of a pool of HuNoVs in children between 1 and 5 years old. Error bars indicated 95% confidence limits.

In participants of the EMaBS longitudinal birth cohort (Figure 1A) seroprevalence to the pool of HuNoV was 76.6% (611/797) by age 1 year, and 94.5%, 95.2%, 96.1%, and 96.6% at 2, 3, 4, and 5 years, respectively (Figure 1B and Supplementary Table 2). Of the sociodemographic variables considered, residence in a rural location (within the EMaBS catchment area) was the only factor significantly associated with having higher antibody levels at age 1 (*P* = .006; Table 1).

**Table 1.** Association Between Human Norovirus (HuNoV) Antibody Levels (OD450 Values) and Sociodemographic Factors Among 797 1-Year-Old Children From the Entebbe Mother and Baby Study Cohort

Factor	Crude GMRs and *P* Values		Adjusted GMRs and *P* Values	
	GMR^a^	*P* Value	GMR^a^	*P* Value
Sex				
Male				
Female	0.99 (0.94–1.04)	.67	0.99 (0.94–1.05)	.78
Location^b^				
Urban				
Periurban	1.06 (1.00–1.13)	.04	1.08 (1.01–1.14)	
Rural	1.09 (1.01–1.17)		1.10 (1.02–1.19)	.02
Mother’s occupation^c^				
Group 1				
Group2	1.00 (0.93–1.07)	.22	1.01 (0.94–1.09)	.19
Group 3	0.94 (0.86–1.03)		0.95 (0.87–1.04)	
Household SES^d^				
1				
2				
3				
4				
5				
6	1.00 (0.98–1.02)	.99 (trend)	1.00 (0.98–1.02)	.99 (trend)
HIV				
Exposed				
Unexposed	0.94 (0.85–1.03)	.14	0.93 (0.85–1.02)	.13
Diarrhea events				
0				
1–2	1.05 (0.99–1.12)	0.12	1.05 (0.99–1.12)	
>2	1.00 (0.92–1.07)		1.00 (0.92–1.07)	.14

All factors were adjusted for each other. Results obtained using linear regression of log10 HuNoV antibody levels and back-transforming to obtain GMRs.

Abbreviation: GMR, geometric mean ratio.

^a^Values in parentheses indicate bootstrapped 95% confidence intervals.

^b^Urban is Entebbe; periurban is Kigungu and Manyago; and rural is Katabi.

^c^Group 1 (farmer/fishing, unskilled manual, and bar/hotel); Group 2 (housewife); and Group 3 (business, student, professional).

^d^SES, household socioeconomic status, 1– 6 (low–high). The GMR for household SES is for each unit increase in SES.

To corroborate the high seroprevalence in the EMaBS cohort and investigate whether it differed in more rural geographical locations, we used a cross-sectional survey (LaVIISWA) conducted in remote island-shore fishing villages in Lake Victoria, only accessible from the mainland in 3 hours by powered canoe (Figure 1A) [18]. Seropositivity to the pool of HuNoVs was higher in 1 year olds in the LaVIISWA survey (94.7%) than in the EMaBS cohort, but was comparable from age 2 onwards (Figure 1B and Supplementary Table 3). Age was the only factor significantly associated with increased antibody levels in this survey (*P* < .0001; Table 2). For every 1-year increase in age, we observed an approximately 5% increase in OD450 readings, suggesting an increase in HuNoV antibody levels that is most likely due to repeat infections (Table 2).

**Table 2.** Association Between Human Norovirus Antibody Levels and Sociodemographic Factors Among 375 Children From the Lake Victoria Island Intervention Study on Worms and Allergy-Related Diseases Survey

Factor	Crude GMRs and *P* Values		Adjusted GMRs and *P* Values	
	GMR^a^	*P* Value	GMR^a^	*P* Value
Sex				
Male				
Female	1.05 (0.98–1.14)	.17	1.08 (0.99–1.16)	.08
Age				
1				
2				
3				
4				
5	1.05 (1.03–1.07)	<.0001 (trend)	1.05 (1.03–1.08)	<.0001 (trend)
Child’s school status				
Student				
Stay at home	1.09 (1.03–1.19)	.04	1.00 (0.92–1.08)	.93

All factors were adjusted for each other. Results obtained using linear regression analyses using the “svy” survey commands in Stata to allow for clustering of respondents within villages using linearized standard errors and for variable village sizes using weights.

Abbreviation: GMR, geometric mean ratio.

^a^Values in parentheses indicate bootstrapped 95% confidence intervals.

**Figure 2.**
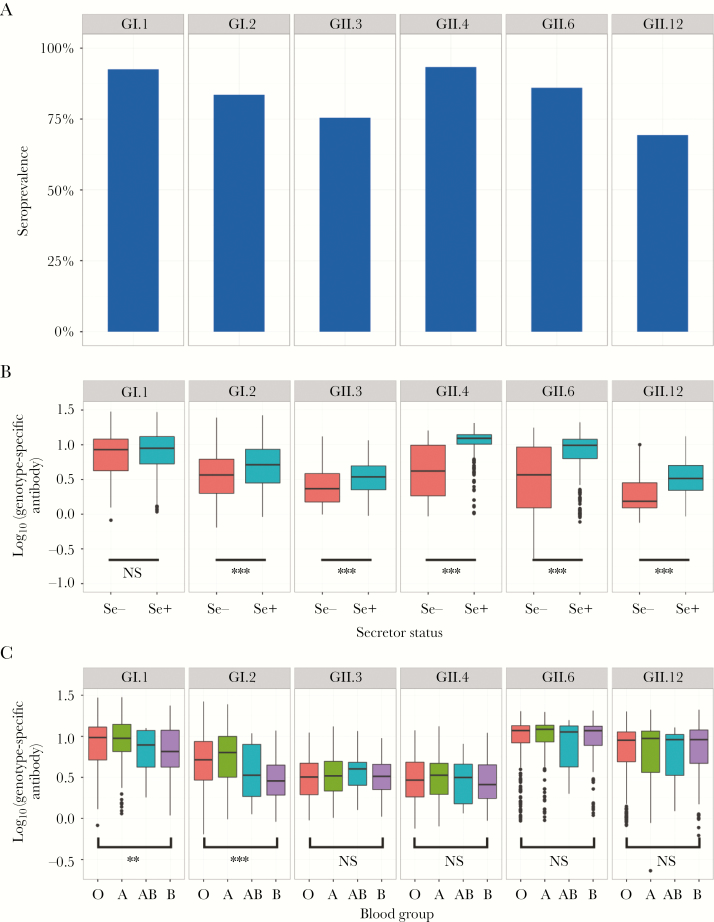
Human genotype analysis of the relationship between human norovirus (HuNoV) antibody levels and α(1,2) fucosyltransferase 2 (FUT2) secretor status and or histo-blood group antigens (HBGA) type. *A*, Seroprevalence of different HuNoV genotypes in 2-year-old participants in the Entebbe Mother and Baby Study. *B*, Association between FUT2 secretor status and genotype-specific antibody levels. *C*, Association between HBGA type and genotype-specific antibody levels. ** *P* < .01; *** *P* < .001; NS, nonsignificant.

**Figure 3.**
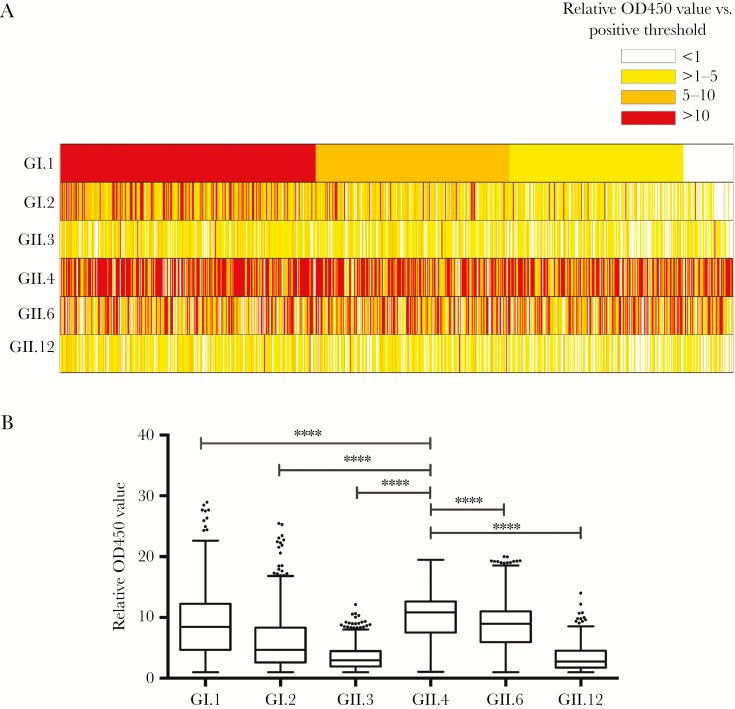
Seropositivity to different human norovirus (HuNoV) genotypes in the 2-year-old age group of a longitudinal clinical cohort of Ugandan children (Entebbe Mother and Baby Study). *A*, The results are represented as a heat map, where each column represents a single sample and are ordered based on GI.1 responses from high to low. Colors indicate the relative OD450 value for each sample against each genotype. *B*, Comparison of the relative OD450 values for each genotype. Boxplot was generated with the Tukey method. Statistically significant differences were analyzed by 1-way ANOVA, using a Dunn multiple comparison test to compare all responses to the highest, GII.4. **** *P* < .0001.

To gain insight into the diversity of HuNoVs circulating in Ugandan children, we determined specific genotype seroprevalence in 2 year olds from the EMaBS cohort. The seroprevalence of the GII.4 virus was comparable to the genotype GI.1 virus at 92.1% and 92.6% respectively (Figure 2A), higher than for all other genotypes (Figure 3). All combinations of genotype responses were observed for different individuals (Figure 3A). Overall, the genotype GII.12 virus had the lowest seroprevalence at 69.4% (Figure 2A). In total, 99.1% of seropositive 2 year olds displayed multiple genotype-specific serological responses.

**Figure 4.**
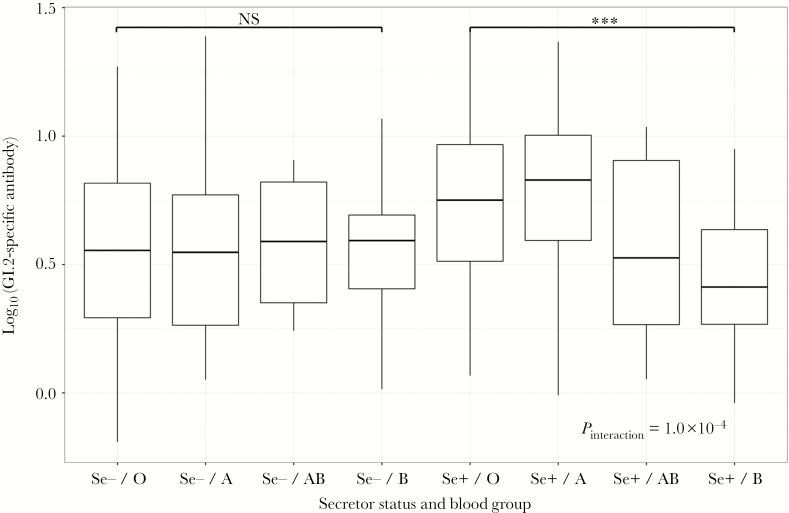
Relationship between α(1,2) fucosyltransferase 2 (FUT2) secretor status, histo-blood group antigens (HBGA) type, and GI.2-specific antibodies. Significance values are provided for tests of differences between distributions in individuals of O or B HGBA type, stratified by secretor status. The final significance value testing for interaction between secretor status and O/B HGBA type is also shown. Distributions of A and AB HGBA type individuals are provided for comparison although not included in the final interaction model. *** *P* < .001; NS, nonsignificant.

As susceptibility to HuNoV-induced diarrhea is at least partially governed by FUT2 secretor (Se) status and gastrointestinal HBGA expression [13], we investigated whether there were associations between levels of genotype-specific antibody and Se− or ABO type in the EMaBS population. Of the 651 EMaBS participants for whom whole-genome genotype data were available, 23.0% (150/651) were Se− (homozygous for the G428A mutation), in line with the frequency previously reported for populations with African ancestry [28]. PCR amplification and Sanger sequencing confirmed the accuracy of secretor status imputation in 80 individuals selected at random. Se− individuals exhibited significantly lower antibody levels against all genotypes except GI.1 (Figure 2B), and the effect was most prominent for GII genotypes, especially GII.4 (geometric mean ratio 1.54 (95% CI, 1.49–1.59); *P* = 3.12 × 10^−52^). In contrast, an effect was only observed at the *ABO* locus for GI genotypes, GI.2 in particular (*P*_omnibus_ = 2.1 × 10^−12^). Given the strong concurrent associations at the *ABO* and *FUT2* loci for GI.2 and the known mechanisms of HBGA expression involving FUT2, we expected to see evidence of epistasis between these 2 loci. We found that Se− individuals had similar distributions of GI.2 specific antibodies irrespective of ABO status and the protective B type effect was only observed in Se+ individuals (*P*_interaction_ = 5.3 × 10^−4^) (Figure 4), as reported in outbreak studies cohorts [29].

Given the high seroprevalence of HuNoV in the Ugandan population, we used the clinical data available within EMaBS to determine the impact of *FUT2* and *ABO* variation on episodes of diarrhea during early childhood. We identified a significantly lower rate of doctor-diagnosed diarrhea in Se− participants <1 year old (hazard ratio 0.89; 95% CI, 0.79–1.00); however, the difference was not sustained to age 5 years. There was statistical evidence for a difference in the association between the 2 age groups (*P* = .008; Table 3). We observed no effect on episodes of diarrhea at the *ABO* locus (Table 3), and the *P* value for the interaction between age and genotype was 0.77.

**Table 3.** Association Between (1,2) Fucosyltransferase 2 (FUT2) Secretor Status and ABO Phenotypic Blood Group and Diarrhea in 0 to 5-Year-Old Children

Age Group	Genotype	Person-Years (× 100)	Number of Diarrhea Events	Rate per 100 person-years	Hazard Ratio (95% CI)	*P* value
Secretor versus nonsecretor (rs601338)						
<1 year	Secretor (AG or GG)	10.9	1657	152.6	1	
	Nonsecretor (AA)	3.1	412	135.1	0.89 (0.79–1.00)	
1–5 years	Secretor (AG or GG)	39.6	1877	47.4	1	.21
	Nonsecretor (AA)	11.3	581	51.5	1.09 (0.95–1.25)	
ABO blood group (rs8176747, rs8176719)						
<1 year	O	7.2	1083	151.3	1	.89
	A	3.6	525	144.2	0.95 (0.84–1.08)	
	AB	0.4	65	144.4	0.95 (0.73–1.24)	
	B	2.7	396	148.9	0.98 (0.87–1.11)	
1–5 years	O	26.3	1289	49.1	1	.77
	A	13.3	639	47.9	0.97 (0.86–1.10)	
	AB	1.6	84	52.8	1.07 (0.76–1.49)	
	B	9.7	446	46.0	0.93 (0.80–1.09)	

## DISCUSSION

Our findings of high seroprevalence in 1-year-old children in the 2 cohorts of Ugandan children exceeds that reported for the same age group from several European countries (for example, 47.3% in Finland, 42.7% in The Netherlands), but becomes comparable by 5 years old [30–32], suggesting greater risk of infection during infancy in Uganda. These findings are consistent with the high incidence reported by RT-PCR–based fecal detection of HuNoVs in community birth cohort studies from India, Ecuador, Peru, and the multicentre MAL-ED study [6, 20, 33, 34]. Such high infection rates may not be captured by incidence reporting in health care settings [7]. Taken as a whole, these community-based studies paint a picture of widespread HuNoV infections occurring early in life in low- and middle-income countries, where children are particularly vulnerable to diarrheal diseases. This suggests that implementation of any future vaccination schedules would need to begin early in life, such as through a birth dose and/or early infant schedule, as per the World Health Organization Expanded Program on Immunization schedule that already exists for routine vaccinations in low-income settings. An effective HuNoV vaccine would also need to confer broad-range protection across genotypes, as is evident from the high percentage of individuals with responses to multiple genotypes by age 2 years.

The seroprevalence was higher in 1-year-old children in the LaVIISWA survey of remote island-shore fishing villages than in the EMaBS cohort. It is possible that this reflects the nature of cross-sectional sampling in LaVIISWA, where those classified as 1 year olds could be closer to age 2, in comparison to the EMaBS where the samples are taken close to each child’s birthday. However the increased seroprevalence was also expected given the lower standards of living in the island communities, including reduced access to proper sanitation and running water [18]. This is in line with our finding that 1-year-old children in rural regions of the EMaBS catchment area had significantly higher antibody levels to the pool of HuNoVs than those in urban areas, suggesting they have a greater chance of recurrent infection.

In estimating the cumulative seroprevalence for the EMaBS longitudinal birth cohort, we had to make a number of assumptions, whilst minimizing the potential of these to lead to overestimation. Firstly, we assumed that samples were missing from the database at random and that the rate of seroconversion for these would be the same as for those which were available in that age group. This would not hold true if the children had different genetic or social risk factors; however, there were no differences in the recorded characteristics for individuals whose samples were present or missing. Secondly, we assumed that once a child had seroconverted they would remain seropositive. This was based on the results of a pilot screen in which we tested consecutive yearly samples from EMaBS participants and found that antibody levels to the pool of HuNoVs remained high each year after seroconversion (Supplementary Figure 1). In support of this, we found that higher antibody levels were significantly associated with increasing age in the LaVIISWA cross-sectional survey, indicative of repeat infections that may boost antibody titers. Additional studies are required to determine whether the IgG responses measured here are protective and capable of neutralizing infection, which could now be tested using the recently developed cell culture systems for HuNoVs [14, 15]. Mucosal IgA has also recently been strongly correlated with protection in human volunteer challenge studies [35] and so inclusion of fecal or salivary IgA responses in any future serological studies will be essential to further understand the duration of protective immune responses.

Surprisingly, considering GII.4 global dominance [36], the seroprevalence of the GII.4 virus was comparable to the genotype GI.1 Norwalk virus. GII.4 antibody levels were, however, significantly higher than for all other genotypes (Figure 3), suggesting either repeat GII.4 infections that boost intragenotype responses, or that the GII.4 virus is more immunogenic, which could be linked to prolonged infections reported for some GII.4 viruses compared to GI viruses [20]. Se− individuals have been shown to be resistant to infection by GI.1 Norwalk virus [37], yet the percentage seropositive for GI.1 exceeded the percentage of Se− individuals in the EMaBS cohort and Se− individuals did not have significantly lower antibody levels to Norwalk virus. As sensitivity to FUT2 status and HBGA expression can vary between strains [13], it is possible that we are measuring intragenotype responses to a related GI.1 virus, which may be able to infect Se− individuals. Previous volunteer studies have also reported a detectable IgG response against Norwalk virus in 78% of Se− individuals, who were otherwise protected from symptomatic infection [37]. Although GI viruses are thought to circulate at low levels worldwide [38], the seroprevalence of GI viruses in some African countries is higher than the global average [9], in line with our serology data. Multiple GI strains have been detected in surface water and sewage in different African countries [9]. Taken altogether, this suggests that GI viruses are endemic in Uganda and unsampled in the community, and that local circulation patterns exist, which may not reflect the global circulation of all strains.

The finding that nearly all seropositive 2 year olds display multiple genotype-specific serological responses, indicates the occurrence of repeat infections and the circulation of diverse HuNoV genotypes, as reported elsewhere [8, 23]. Importantly, the possibility of cross-reactive antibody responses, particularly between genotypes of the same genogroup, such as GII.4 and GII.6, cannot be excluded. However, we observed all combinations of genotype responses, strongly supporting assay specificity, and limited cross-reactivity has been reported for children <5 years old [39]. Furthermore, the genotypes used here have all recently been proposed to fall into different “immunotypes,” reducing the likelihood of cross-reactive responses [23]. Several African case-control studies have described relatively high detection rates of HuNoV secretion in healthy controls [9]. It remains unclear whether this is due to asymptomatic infection or recent resolution of symptomatic infection; however, these studies suggest that the appearance of symptoms may vary with genotype, host susceptibility (potentially Se status), and acquired immunity. Further incidence studies are therefore required to confirm which circulating genotypes cause the most substantial disease in Uganda and how this links to Se status in this population.

In the context of numerous reports of incidence in health care settings [8–10], this dataset provides essential insight into the high community prevalence of HuNoVs in children in sub-Saharan Africa and suggests that HuNoV infections are ubiquitous, recurrent, occur within the first year of life, and may be a substantial cause of endemic childhood diarrhea in Uganda. Other birth cohort studies have shown that multiple symptomatic HuNoV infections at an early age are associated with growth stunting [20]. This raises concerns as to whether the commonality of multiple HuNoV infections could be impacting the growth and development of young Ugandan children and further studies will also be important to address this. Our data support the need for public health interventions coupled with routine HuNoV surveillance and molecular diagnostics across Africa, the latter of which could be achieved by expansion of the African Rotavirus Surveillance Network to include HuNoVs. This is particularly important given the high prevalence of unsampled community HuNoV infections suggests that emergence of novel variants in Africa is likely.

## Supplementary Data

Supplementary materials are available at *The Journal of Infectious Diseases* online. Consisting of data provided by the authors to benefit the reader, the posted materials are not copyedited and are the sole responsibility of the authors, so questions or comments should be addressed to the corresponding author.

Supplementary Material

Supplementary Methods

Supplementary Figure 1

## Notes

***Acknowledgments.*** We thank Edward Emmott for his technical assistance in preparing VLPs.

***Financial support.*** This work was supported by a Sir Henry Wellcome Fellowship to L. T., and senior fellowships to I. G. and A. E.; a European Research Council Advanced Grant to A. H. (grant number 294557); and a Wellcome Trust Clinical Research Training Fellowship to A. M. (grant number 106289). Entebbe Mother and Baby Study and Lake Victoria Island Intervention Study on Worms and Allergy-related diseases are funded by the Wellcome Trust (grant numbers 064693, 079110, 95778).

***Potential conflicts of interest.*** All authors: No reported conflicts of interest. All authors have submitted the ICMJE Form for Disclosure of Potential Conflicts of Interest. Conflicts that the editors consider relevant to the content of the manuscript have been disclosed.
