# Supplementary material for: The First Norovirus Longitudinal Seroepidemiological Study From Sub-Saharan Africa Reveals High Seroprevalence of Diverse Genotypes Associated With Host Susceptibility Factors
Source: J Infect Dis. 2018 Apr 18;218(5):716–25. doi: 10.1093/infdis/jiy219 (PMC6057498; doi:10.1093/infdis/jiy219)
Supplement: Supplementary Material [file jiy219_suppl_supplementary-material.docx]

**Supplementary material:**

**Supplementary Figure 1**. IgG responses to HuNoVs over time for five EMaBS participants. Samples each year after from five randomly selected EMaBS participants were screened for IgG responses to a pool of HuNoV VLPs. Samples were missing for child 4 at year 4 and 5. Each sample was screened in technical triplicates, error bars represent the standard deviation.

**Supplementary Tables**

**Supplementary Table 1. Determination of genetic blood group from phased SNPs. The phased base on the positive strand and corresponding genetic group is shown for each chromosome.**

|  | rs8176719 (chromosomes 1:2) | | | |
| --- | --- | --- | --- | --- |
| rs8176747 (chromosomes 1:2) | T:T | T:TC | TC:T | TC:TC |
| C:C | OO | OA | AO | AA |
| C:G | OO | OB | AO | AB |
| G:C | OO | OA | BO | BA |
| G:G | OO | OB | BO | BB |

**Supplementary Table 2. Summary of the sample size and the cumulative seroprevalence of HuNoV in the EMaBS longitudinal clinical cohort of Ugandan children.**

| Age | n | m | a | Pos | Neg | SC rate |  | Est. pos | Cum.SP | 95% CI |
| --- | --- | --- | --- | --- | --- | --- | --- | --- | --- | --- |
| 1 | 797 | - | 797 | 611 | 186 | 0.767 |  | 611 | 76.6 | 73.6 – 79.6 |
| 2 | 186 | 76 | 110 | 84 | 26 | 0.764 |  | 142 | 94.5 | 92.7 – 96.0 |
| 3 | 26 | 4 | 22 | 5 | 17 | 0.277 |  | 5.9 | 95.2 | 93.5 – 96.6 |
| 4 | 17 | 7 | 10 | 4 | 6 | 0.4 |  | 6.8 | 96.1 | 94.5 – 97.3 |
| 5 | 6 | - | 6 | 4 | 2 | 0.667 |  | 4 | 96.6 | 95.1 – 97.8 |

n: number of children. m: number of missing samples. a: Number of available samples. Pos: actual number that tested positive. Neg: actual number that tested negative. SC rate: rate of seroconversion. Est pos: estimated positive numbers, calculated by applying the seroconversion rate observed in available samples to the total number of children who tested negative at the previous time point. This was used to calculate cumulative seroprevalence as described in Methods.Cum. SP: Cumulative seroprevalence. CI: confidence intervals.

**Supplementary Table 3. Seroprevalence of HuNoV infection in a cross-sectional clinical survey of children (LaVIISWA) living in island fishing communities around Lake Victoria.**

| Age Group | Sample Size | Seropositivity (%) | 95% Confidence Interval |
| --- | --- | --- | --- |
| 1 | 94 | 95.2 | 87.1 – 98.3 |
| 2 | 89 | 95.3 | 83.4 – 98.8 |
| 3 | 76 | 97.3 | 89.8 – 99.3 |
| 4 | 65 | 100 | - |
| 5 | 54 | 99.1 | 92.8 – 99.9 |
