# Supplementary material for: The First Norovirus Longitudinal Seroepidemiological Study From Sub-Saharan Africa Reveals High Seroprevalence of Diverse Genotypes Associated With Host Susceptibility Factors
Source: J Infect Dis. 2018 Apr 18;218(5):716–25. doi: 10.1093/infdis/jiy219 (PMC6057498; doi:10.1093/infdis/jiy219)
Supplement: Supplementary Methods [file jiy219_suppl_supplemental_methods.docx]

**Supplemental Methods**

**VLP strain selection**

The reported seroprevalence for each genotype will relate particularly to the strain selected, although it is likely that cross-reactive intra-genotype responses to related strains will also be detected, depending on the degree of epitope conservation. For non-GII.4 genotypes (GI.2, GII.3, GII.6 and GII.12) that are thought to be more antigenically static [1, 2], we selected strains that had emerged during the EMaBS sampling period, hypothesising that these would allow us to detect intra-genotype responses to old and new related strains. However it is possible that this may result in lower rates of detection of these genotypes. Conversely, GII.4 are highly variable antigenically [3] so it is possible that using an older strain may not capture responses to newer GII.4 strains.

**ELISA for HuNoV IgG Levels**

For the ELISAs, one plasma sample was screened per participant at each age. Participants were selected randomly from the EMaBS cohort by assigning random numbers to every 1-year-old sample in the database using Stata. We confirmed there were no duplicates in the randomly generated numbers, sorted them in ascending size order and selected the top 800 samples, of which 3 were missing. Plasma samples were diluted 1:50 and screened in duplicate against the pool of HuNoV VLPs (comprised of 2.5ng of each VLP per well) in the first instance and later individual genotypes (12.5ng per well). The background signal for each sample was calculated using 2 blank wells, the average of which was subtracted from the average OD450 value for each sample to give the corrected OD450. A positive control (human pooled sera, Sigma Aldrich) was included on each plate. The threshold for a positive response was determined as the average of all blanks on the plate, plus three standard deviations, as previously established [4]. To be able to compare responses across different genotypes and across different plates, the relative OD450 value was calculated as the fold difference between each sample and the positive threshold.

**Statistical analysis**

To confirm associations between secretor status and ABO phenotypic blood group and IgG levels, sensitivity analyses were performed to ensure that the statistics generated from the genetic association analyses were not false positives originating from population stratification. Firstly, linear regression models were used in place of the t-test adapted to include the first ten genetic principal components. These components were calculated using the EIGENSOFT software v4.2 [5] with autosomal variants pruned by linkage disequilibrium (r2<0.5) and removing regions of the genome under long-range linkage disequilibrium (such as the human leukocyte antigen complex). Inclusion of the first ten principal components did not adversely affect the strength of association for any tested secretor or ABO variants. Furthermore, we implemented a linear mixed model method of association testing that includes a genetic relatedness matrix (calculated using all autosomal SNP data) to explicitly model relationships between all individuals as a random effect covariate in matrix format. These results were generated using the GEMMA software [6], and were nearly identical to the model including principal components alone.

References

1. Parra, G.I., et al., *Static and Evolving Norovirus Genotypes: Implications for Epidemiology and Immunity.* PLoS Pathog, 2017. **13**(1): p. e1006136.

2. Boon, D., et al., *Comparative Evolution of GII.3 and GII.4 Norovirus over a 31-Year Period.* J Virol, 2011. **85**(17): p. 8656-8666.

3. Bull, R.A., et al., *Rapid Evolution of Pandemic Noroviruses of the GII.4 Lineage.* PLoS Pathog, 2010. **6**(3): p. e1000831.

4. Caddy, S., et al., *Serological Evidence for Multiple Strains of Canine Norovirus in the UK Dog Population.* PLoS ONE, 2013. **8**(12): p. e81596.

5. Price, A.L., et al., *Principal components analysis corrects for stratification in genome-wide association studies.* Nat Genet, 2006. **38**(8): p. 904-9.

6. Zhou, X. and M. Stephens, *Genome-wide efficient mixed-model analysis for association studies.* Nat Genet, 2012. **44**(7): p. 821-4.
