# Supplementary figures and images for: The First Norovirus Longitudinal Seroepidemiological Study From Sub-Saharan Africa Reveals High Seroprevalence of Diverse Genotypes Associated With Host Susceptibility Factors
Source: J Infect Dis. 2018 Apr 18;218(5):716–25. doi: 10.1093/infdis/jiy219 (PMC6057498; doi:10.1093/infdis/jiy219)

### Seroconversion against HuNoV

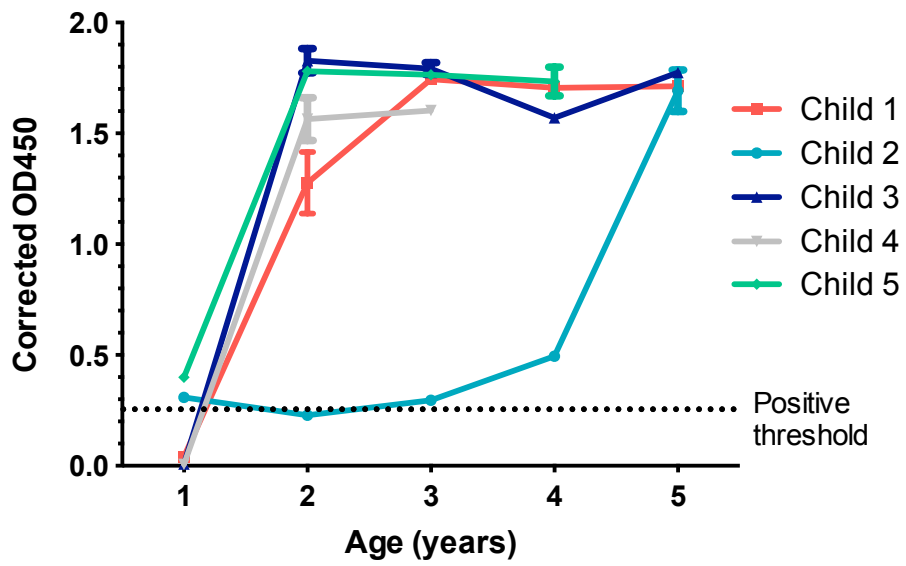

Supplement: Supplementary Figure 1 [file jiy219_suppl_supplemental_figure.pdf]
